# Unraveling the activity of phage-carrying antibiotic resistance genes in constructed wetlands

**DOI:** 10.3389/fcimb.2026.1764958

**Published:** 2026-02-09

**Authors:** Qian Zhao, Donglin Wang, Hui Lin, Tong Zhou, Jun Zhang, Jiayu Shang, Dehan Cai, Yanni Sun, Zhen Hu, Jian Zhang

**Affiliations:** 1 Shandong Key Laboratory of Synergistic Control of Complex Multi-Media Pollution, School of Environmental Science & Engineering, Shandong University, Qingdao, China; 2 State Key Laboratory of Microbial Technology, Shandong University, Qingdao, China; 3 Shandong Provincial Key Laboratory of Animal Cell and Developmental Biology, School of Life Sciences, Shandong University, Qingdao, China; 4 Advanced Interdisciplinary Institute of Environment and Ecology, Guangdong Provincial Key Laboratory of Wastewater Information Analysis and Early Warning, School of Technology for Sustainability, Beijing Normal University, Zhuhai, China; 5 Department of Information Engineering, Chinese University of Hong Kong, Hong Kong, Hong Kong SAR, China; 6 Department of Electrical Engineering, City University of Hong Kong, Hong Kong, Hong Kong SAR, China

**Keywords:** antimicrobial resistance, deep learning, horizontal gene transfer, metagenome, metatranscriptome

## Abstract

Antimicrobial resistance (AMR) is a global public health challenge, and risk assessments based solely on gene abundance often underestimate the immediacy of resistance dissemination. This study presented a carrier-centric framework integrating metagenomic and metatranscriptomic profiling with deep learning–based identification of mobile genetic elements, applied to a full-scale constructed wetland (CW). CW overall reduced ARG burdens, with genomic abundance in plants, sediments, and water decreasing by 98.5%, 80.9%, and 88.8%, respectively. However, transcriptional activity showed an opposite trend, with sediments exhibiting the highest ARG expression, highlighting their pivotal role in the persistence and dissemination of resistance. In sediments, phage-mediated expression increased sharply from 4.0% to 92.5%, exceeding plasmid-associated levels by ~276-fold, revealing a low-abundance but high-activity residual risk pattern. Furthermore, 16 of the 310 recovered nonredundant MAGs were identified as phage hosts, 11 of which were potentially pathogenic, antibiotic-resistant bacteria (PARB) and were more active in sediments than in water or plants. These findings indicate that transduction within high-density, biofilm-associated niches constitutes a key terminal risk source. In addition, sediment acts as a high-risk reservoir where redox and ionic gradients, together with residual lomefloxacin and other antibiotics, enhance phage infectious activity and the accumulation of ARGs. Through cross-compartment transmission along the sediment–water interface, these phage-associated and PARB populations continuously seed the overlying water. It is recommended that ARG risk assessment shift from static abundance to an activity-aware, carrier- and host-resolved approach, prioritizing sediment-targeted transcript monitoring and phage transduction early warning to support risk mitigation in CW.

## 1 Introduction

Antimicrobial resistance (AMR) poses a substantial threat to human health, and the presence of antibiotic resistance genes (ARGs) is the root cause of bacterial resistance. In recent decades, the increasing abundance of ARGs in the environment and the spread of ARGs have attracted growing attention due to their significant public health risks (Chen et al., 2019). The World Health Organization identifies AMR as a defining public health challenge of the twenty-first century (Liu et al., 2022). Horizontal gene transfer (HGT) denotes the transfer of genetic material between organisms outside of parent–offspring descent and is widely regarded as a cornerstone of bacterial evolution (Soucy et al., 2015; Dmitrijeva et al., 2024). Because HGT enables genes to bypass lineage constraints, the environmental risk posed by ARGs primarily stems from their capacity to spread across hosts and settings, exemplified by the mobilization of resistance determinants from commensals to pathogens (Karkman et al., 2018). The dynamics of ARGs are shaped not only by antibiotics but also by non-antibiotic factors, including nutrient regimes and heavy metals, which can promote the production, maintenance, and enrichment of ARGs through selection and co-selection (Shi et al., 2022). Among HGT pathways, plasmid-mediated conjugation and phage-mediated transduction are the principal routes for the transmission of ARGs (Liu et al., 2024a; Wang et al., 2024a). Studies have predicted that if effective interventions are not implemented on time, the global spread of ARG may cause at least 10 million deaths each year by 2050 (Jiang et al., 2025). Consequently, mitigating ARG risks requires the precise identification and tracking of these mobile genetic elements (MGE)—especially plasmids and phages—to decipher, monitor, and ultimately interrupt the spread of ARGs.

HGT in the environment could be captured by culture-dependent isolation or reporter gene technology to establish the occurrence of transfer and to quantify its transfer frequency. For example, Zhao et al. (2023) added donor *E. coli* carrying the plasmid to compare conjugative transfer under micro- and nano-plastic exposures of varying particle sizes and quantified the resulting transfer frequencies. By concentrating on a limited set of representative MGEs and a restricted recipient (host) range, and given that many environmental bacteria are not cultivable, these approaches probably underestimate the true transfer frequency of ARGs. As a typical culture-independent technology, metagenomics (metaG) overcomes many of these constraints. It has been widely used to characterize community structure and functional pathways in various ecosystems such as soil, rivers, and sewage treatment, as well as to quantify ARGs and MGEs (Zhang et al., 2025b; Zhu et al., 2025). However, alignment-based MGE identification is limited by the lack of universal phage markers, the highly mosaic nature of plasmid genomes, and poorly defined plasmid–chromosome boundaries, which complicate threshold calibration and increase false-negative rates, ultimately lowering the overall recall of plasmid and phage detection (Wang et al., 2024a). Deep-learning (DL) based methods promote the identification of MGEs, especially phages and plasmids, by learning high-dimensional sequence features beyond simple homology. They improve detection of low-identity or short plasmid/phage contigs and enable precise delineation and excision of prophages from host genomes, thereby lowering false-negative rates and sharpening MGE boundary resolution and host assignment (Arango-Argoty et al., 2018). Furthermore, key ARGs can be linked to hosts using metagenome-assembled genomes (MAGs), enabling the construction of an ARG–MGE–host association network (Zhao et al., 2020). Together, metaG and DL-based classifiers provide a scalable framework to map HGT events and more accurately resolve the plasmid and phage vehicles that disseminate ARGs.

Measuring transmission risk solely by abundance may underestimate or misjudge the immediate threat posed by ARGs (Klumper et al., 2025). Transcriptional activity offers a closer view of function, revealing which ARGs are being expressed and potentially mobilized (Liu et al., 2019). Under the framework of the central dogma, phage-associated ARG expression may reflect phage–host interactions and could be linked to horizontal ARG transfer. Therefore, metatranscriptomics (metaT) is a valuable tool for detecting functionally expressed transcripts that evaluate transcripts across all microbes within a community (Li et al., 2025). MetaT also faces practical challenges, including RNA degrades readily and is highly sensitive to sampling and storage conditions, and relying solely on *de novo* metaT assembly can overlook low-abundance or short transcripts while hindering accurate assignment of transcriptional signals to specific hosts (e.g., plasmids or phages) (Ojala et al., 2023). By contrast, metaG generally provides broader community coverage. In recent years, studies have constructed metagenomic references (contigs/MAGs) and mapped metaT reads back to them to obtain host-resolved expression of target genes (e.g., ARGs) for ARG expression assessment (Mohamed et al., 2025). By integrating metaG with metaT, it becomes possible to jointly capture the genomic potential and transcriptional activity of ARGs, while enabling carrier-resolved and activity-aware evaluation of ARG dissemination risk that cannot be achieved by either approach alone. However, the activity of plasmids and phages in driving ARG dissemination remains poorly resolved.

Building on the need for activity-aware and MGE-resolved measurements of ARGs, we use constructed wetlands (CWs) as a tractable model system to study ARG active dynamics and horizontal transfer. CWs, a nature-based technology for wastewater treatment, are widely adopted for their low operating costs, simple operation and maintenance, and environmental sustainability, and are increasingly deployed as advanced wastewater-treatment technology (Zhao et al., 2025). As the last line of defense against contaminant discharge to aquatic environments, CWs are typically positioned between wastewater treatment plants (WWTPs) and receiving waters (Karstens et al., 2016). Because WWTPs were originally designed and managed to remove conventional constituents, their capacity to abate ARGs is comparatively limited, allowing ARGs to persist in effluents and be subsequently introduced into downstream CWs (Wang et al., 2024b). Additionally, the high microbial densities, well-developed biofilms, and complex physicochemical gradients in CWs may render them hotspots for the selection, enrichment, and dissemination of ARGs (Zhao et al., 2024). Specifically, the presence of antibiotic residues and co-occurring contaminants in CWs may impose selective and stress pressures on microbial communities, potentially activating stress responses such as the SOS response that are known to promote HGT (Beaber et al., 2004). In parallel, plant-mediated processes, including root exudation and rhizosphere effects, can reshape microbial community composition and local selection pressures, thereby influencing ARG enrichment and dissemination (Guan et al., 2025). For example, Zhao et al. (2024) extensively characterized the abundance and composition of ARGs and their bacterial hosts, providing important insights into the structure and distribution of resistomes; however, such analyses are largely limited to DNA-level signals and cannot reflect functional activity. Similarly, Calero-Caceres et al. (2016) further distinguished ARGs associated with different mobile genetic elements, yet these approaches still lack resolution of transcriptional activity and are unable to determine whether detected ARGs are actively expressed or mobilized. Consequently, current monitoring frameworks remain predominantly static and gene-centric, underscoring the need for activity-aware and MGE-resolved strategies to more accurately assess ARG dissemination risk in engineered ecosystems such as constructed wetlands.

Here, we move beyond static, gene-centric surveys to a carrier-centric, activity-aware view of ARG dissemination in full-scale CW by integrating coordinated metaG–metaT analysis. Specifically, we (i) apply DL-based methods to delineate plasmid and phage sequences—and prophage boundaries—in metagenomic assemblies; (ii) map metatranscriptomic reads to quantify ARG transcription on these MGE backbones; (iii) link MGEs to hosts via MAGs; and (iv) integrate these signals into an HGT activity that couples ARG expression on MGEs with indicators of mobilization. Through the application of this method, we were able to utilize metaG and metaT datasets from full scale CWs to characterize the distributional patterns of ARGs. By resolving the active plasmids and phages—and their hosts—this framework moves beyond presence or bulk abundance to identify the vehicles currently propagating resistance *in situ*, providing a direct readout of real-time transmission risk. The resulting, carrier- and host-resolved assessment provides a practical basis for prioritized surveillance and management in CWs and a generalizable blueprint for activity-aware ARG risk evaluation across engineered and natural ecosystems.

## 2 Materials and methods

### 2.1 Sample collection and pre-processing in a full-scale constructed wetland

**Figure 1:**
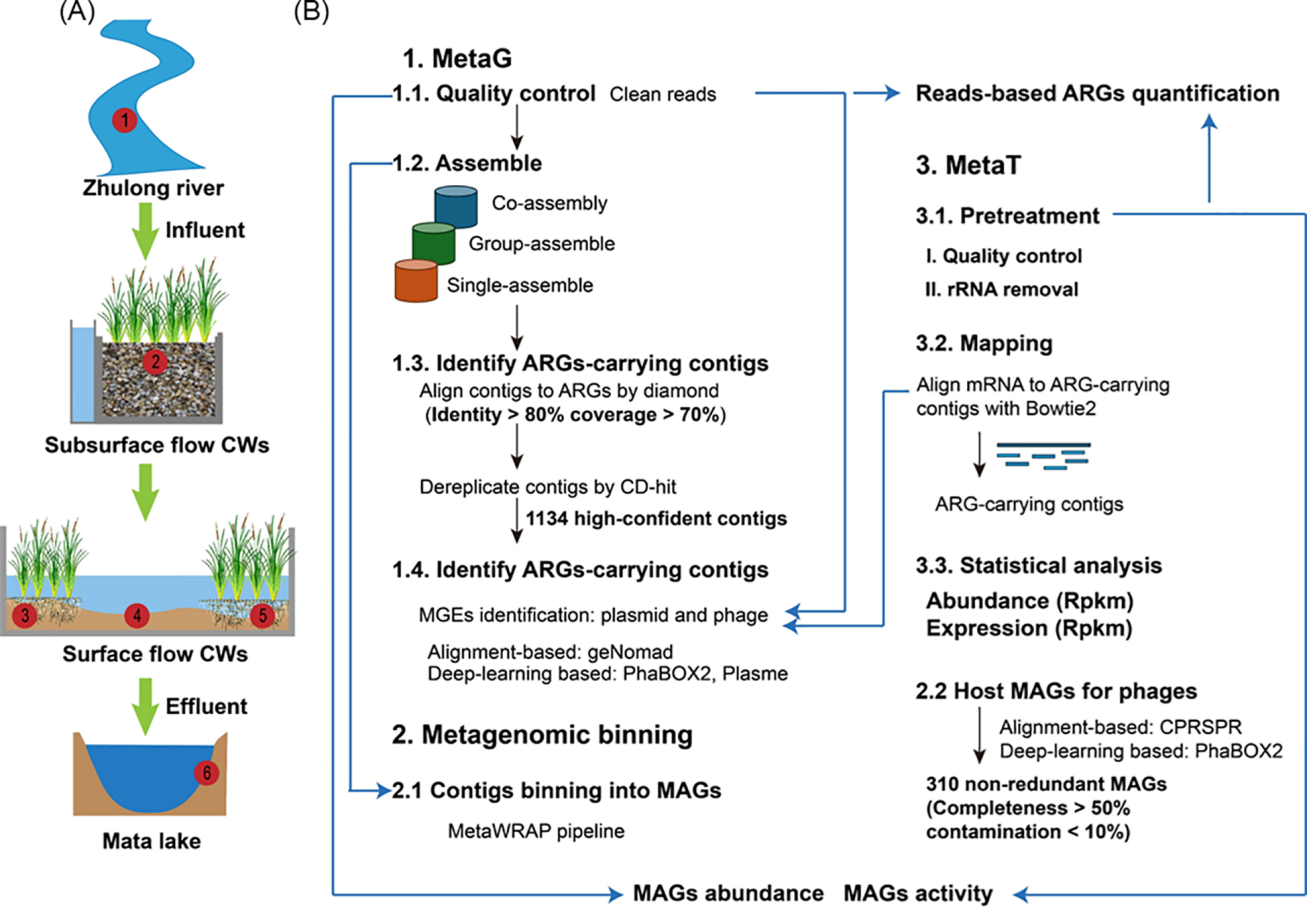
Sampling layout and an integrated metagenomic/metatranscriptomic workflow for the CWs. a Sampling sites. Six sites along the treatment train: M1 influent from the Zhulong River; M2, **(A)** horizontal subsurface-flow wetland; M3-M5, surface-flow wetland cells; and M6, effluent; and **(B)** Integrated meta-omics workflow.

Mata Lake CW is located in Zibo City, Shandong Province, China, and primarily treats wastewater from the Zhulong River, which is affected by agricultural runoff, domestic sewage, and industrial effluents. The Mata Lake CW covers 47.6 hectares and operates at an average throughput of 20,000 m³ d^−1^. Mata Lake CW is a typical hybrid CW consisting of an ecological retention pond (1.33 hectares), a horizontal subsurface-flow wetland (3.67 hectares), and a large surface flow wetland (42.6 hectares). In October 2021, six sites were strategically distributed across the Zhulong River (influent), horizontal subsurface-flow CWs, surface-flow CWs, and the effluent (**Figure 1A**). This spatial arrangement represents the core functional treatment units of the CWs, from inflow to discharge. A total of 16 samples were collected from Mata Lake CW, including six water samples (MW1–MW6), six sediment samples (MS1–MS6), and four plant samples (MP2–MP5). At each sampling site, 5 liters (L) of surface water were collected in pre-sterilized high-density polyethylene bottles, carefully avoiding sediment resuspension. 1L was reserved for water-quality analyses and was kept in the dark at 4 °C. The remaining 4 L were vacuum-filtered on site through pre-rinsed 0.22 µm polycarbonate membranes (MilliporeSigma, USA) using a portable vacuum filtration pump (Sciencetool, China). Following filtration, membranes were aseptically removed using sterile forceps and placed into sterile 50 mL centrifuge tubes. Sediment samples (5–10 cm deep) from the CWs were collected with a Petite Ponar grab (Wildlife Supply Company, USA) in three independent replicates per site. Plants (*Acorus calamus*) were manually excavated, washed to remove loosely attached sediment, and stored in sterile bags. Plant was defined as the plant-associated microbiota, including the rhizosphere and plant endophytic microbiome in our manuscript. All samples were stored at −80 °C for subsequent analysis. To minimize cross-contamination among sample types, reusable tools were decontaminated with 70% ethanol between samples.

To systematically evaluate the spatial trajectories and dissemination potential of ARGs and their genetic carriers along the continuous “source–CW–receiving water” gradient, paired metaG and metaT datasets were generated for all samples and processed as outlined in **Figure 1B**. Following pretreatment, total DNA was extracted from the water and sediment samples using the DNeasy PowerSoil Kit (QIAGEN, Germany). For the plant samples, tissues were washed with sterile water before DNA extraction using the DNeasy Plant Maxi Kit (QIAGEN, Germany). The extracted DNA was purified with the DNeasy PowerClean CleanUp Kit (QIAGEN, Germany), and the DNA purity was assessed using a Nano-Photometer (Implen, Westlake Village, CA). High-quality DNA was used for library construction and submitted to Biozeron Biological Technology Co., Ltd. (Shanghai, China) for metagenomic sequencing on the Illumina NovaSeq 6000 platform.

Total RNA was extracted from pretreated water, sediment, and plant samples using the RNeasy Mini Kit (QIAGEN, Germany). Concentrated RNA was reverse-transcribed into cDNA using the SuperScript^®^ III First-Strand Synthesis System (Invitrogen, USA) with random hexamer primers, followed by second-strand synthesis with the SuperScript^®^ Double-Strand cDNA Synthesis Kit (Invitrogen, USA). The resulting cDNA libraries were used for metatranscriptomic sequencing, which was conducted on the Illumina NovaSeq 6000 platform by Biozeron Biological Technology Co., Ltd. (Shanghai, China). All metagenomic and metatranscriptomic sequencing reads have been submitted to the National Center for Biotechnology Information (NCBI) database under the accession number PRJNA1378718.

The *in-situ* water quality parameters, such as temperature (T), pH, oxidation-reduction potential (ORP), and dissolved oxygen (DO), were measured using a portable multi-parameter probe (Multi 3430 WTW, Germany). The water’s other quality parameters, including chemical oxygen demand (COD), NH_4_^+^-N, NO_3_^--^N, and total nitrogen (TN) were measured using standard methods (APHA, 2012). Sediment T, ORP, and EC were measured *in situ* using a portable multi-parameter sonde. Sediment pH was determined by mixing 10 g of dried, sieved, and homogenized sediment with 25 mL of ultrapure water; after standing for 30 min, pH was measured using a calibrated pH probe. Sediment samples were dried at 37 °C to constant weight and passed through a 100-mesh soil sieve. The powdered samples were extracted with 1 mol L^−1^ KCl at a ratio of 1:5 (w/v), and NH_4_^+^-N, NO_3_^--^N, and TN were determined with reference to a previous study (Liu et al., 2024b). Total organic carbon (TOC) in sediment samples was determined using a TOC analyzer (TOC-L, Shimadzu, Japan). Detailed results were shown in **Supplementary Tables S3** and **S4**.

### 2.2 Bioinformatic analysis

#### 2.2.1 Metagenomic assembly and genome binning

The raw (metaG) reads were trimmed utilizing fastp (v1.0.1) (Chen et al., 2018). Trimmed reads were assembled using MEGAHIT (v1.2.9) (Li et al., 2015) with the “meta-large” preset parameter by three assembly strategies: single, grouped, and co-assemble. Accordingly, contigs greater than 3000 bp were binned into draft genomes using MetaWRAP (Uritskiy et al., 2018) with default parameters. All sets of bins were subsequently refined using the “bin_refinement” module in MetaWRAP to achieve optimized MAGs. Quality assessment of all MAGs, encompassing completeness, contamination, and heterogeneity, was conducted using CheckM (Parks et al., 2015). Bins with completeness <50% or contamination ≥10% were removed from subsequent analysis. All bins were dereplicated at 95% average nucleotide identity (species level) to obtain non-redundant MAGs using dRep (v3.4.5) (Olm et al., 2017).

#### 2.2.2 Taxonomic assignment and phylogenetic inference of assembled MAGs

The taxonomic assignment of all MAGs was determined using GTDB-Tk (v2.3.2) with reference data version r207 (Chaumeil et al., 2019). Subsequently, the MAGs were selected for phylogenomic tree construction. Briefly, multiple sequence alignments of 53 and 120 concatenated conserved marker genes for Archaea and Bacteria were obtained from GTDB-Tk, respectively. Poorly aligned regions were removed using trimAl (v1.4.rev22) with the parameter: -automated (Capella-Gutiérrez et al., 2009). Maximum-likelihood phylogenetic trees were constructed using IQ-TREE (v1.6.12), with 1,000 ultrafast bootstrapping iterations (Minh et al., 2020). Final trees were visualized using iTOL (Letunic and Bork, 2019).

#### 2.2.3 ARGs identification

Read-based annotation and quantification of ARGs was performed on trimmed reads of each sample using ARG-OAP (v3.2.2) (Yin et al., 2023) with a cut-off of similarity of 80%, query length coverage ratio of 75%, and e-value of 1e-7. The abundance of potential ARGs in each sample was normalized based on cell numbers (ARG copies per cell) at the “type” level according to previously reported methods (Liang et al., 2020; Chen et al., 2022). To obtain contig-resolved ARGs, assembled contigs from all three datasets were dereplicated with CD-HIT v4.7 at 100% local similarity (Fu et al., 2012). ARGs were then annotated by DIAMOND v2.1.6 (blastx) against the SARG protein database bundled with ARG-OAP (Buchfink et al., 2015), requiring ≥80% identity and ≥70% alignment coverage. ARGs were also identified in MAGs with the same method. Additionally, pathogenic MAGs were also identified by the PHI database (Urban et al., 2024). Contigs containing at least one ARG were designated as ARG-carrying contigs. Genetic mobility was assessed next. Phage-borne ARG-carrying contigs were identified by integrating alignment-based method, geNomad and deep-learning predictions from PhaMer (Shang et al., 2022; Camargo et al., 2023). Only the temperate phage identified by PhaBOX was further analyzed. Plasmid-borne ARGs were identified with PLASMe (Tang et al., 2023) (DL based). Contigs lacking phage or plasmid signatures were treated as chromosomal (non-mobile) ARG carriers. Further, hosts of ARG-carrying phages were inferred using the CHERRY module in PhaBOX2 (Shang et al., 2023).

#### 2.2.4 Abundance and expression quantification

Metagenomic abundance was estimated by mapping quality-trimmed reads from each sample to ARG-carrying contigs and dereplicated MAGs using CoverM v0.6.1 in contig and genome modes, respectively, with identical settings: --min-read-percent-identity 0.95, --min-read-aligned-percent 0.75, and --methods rpkm. Metatranscriptomic expression was quantified after preprocessing raw reads with fastp and removing residual rRNA with SortMeRNA v2.1 (Kopylova et al., 2012). The remaining mRNA reads were then competitively mapped to the same MAGs and ARG-carrying contigs using CoverM with the parameters listed above, producing RPKM values directly comparable to the metaG estimates.

#### 2.2.5 Network construction

Co-occurrence networks among different types of ARGs were constructed based on pairwise associations between their normalized abundance and expression levels. Spearman’s rank correlation coefficients were calculated using the *psych* package in R to evaluate co-variation patterns among ARG categories. To control for multiple testing, *P*-values were adjusted using the false discovery rate (FDR) procedure. Edges were retained only when correlations met both criteria: |Spearman ρ| ≥ 0.8, and FDR-corrected P < 0.05. Network visualization and characterization—including degree distribution, network diameter, node connectivity, and clustering coefficients—were performed in Gephi (version 0.10.1) using default analytical modules.

The bipartite network was constructed to represent the connectivity between samples of different compartments (Plant, water, and sediment) and MAGs based on expression. An edge was defined when the RPKM of a MAG in a given sample exceeded a threshold of RPKM≥ 1, which was considered evidence of presence and potential ecological interaction. The network graph (g) was generated using the *tidygraph* and *ggraph* frameworks in R.

### 2.3 Statistical analysis

All statistical analyses were performed in R (v 3.6.2). In all experiments, differences and correlations were considered statistically significant when *p* < 0.05. Pairwise distances between each environmental factor (including temperature, pH, ORP, COD, NH_4_^+^-N, NO_3_^--^N, TN) were computed using the ggcor package in R. To identify the relationship between the composition of phage, pathogenic antibiotic-resistant bacteria, and host MAGs of ARG-carrying phage and environmental factors, partial Mantel correlations were computed (9999 permutations) using the ggcor package in R.

## 3 Results and discussion

### 3.1 ARG trajectories across the CW treatment process

**Figure 2:**
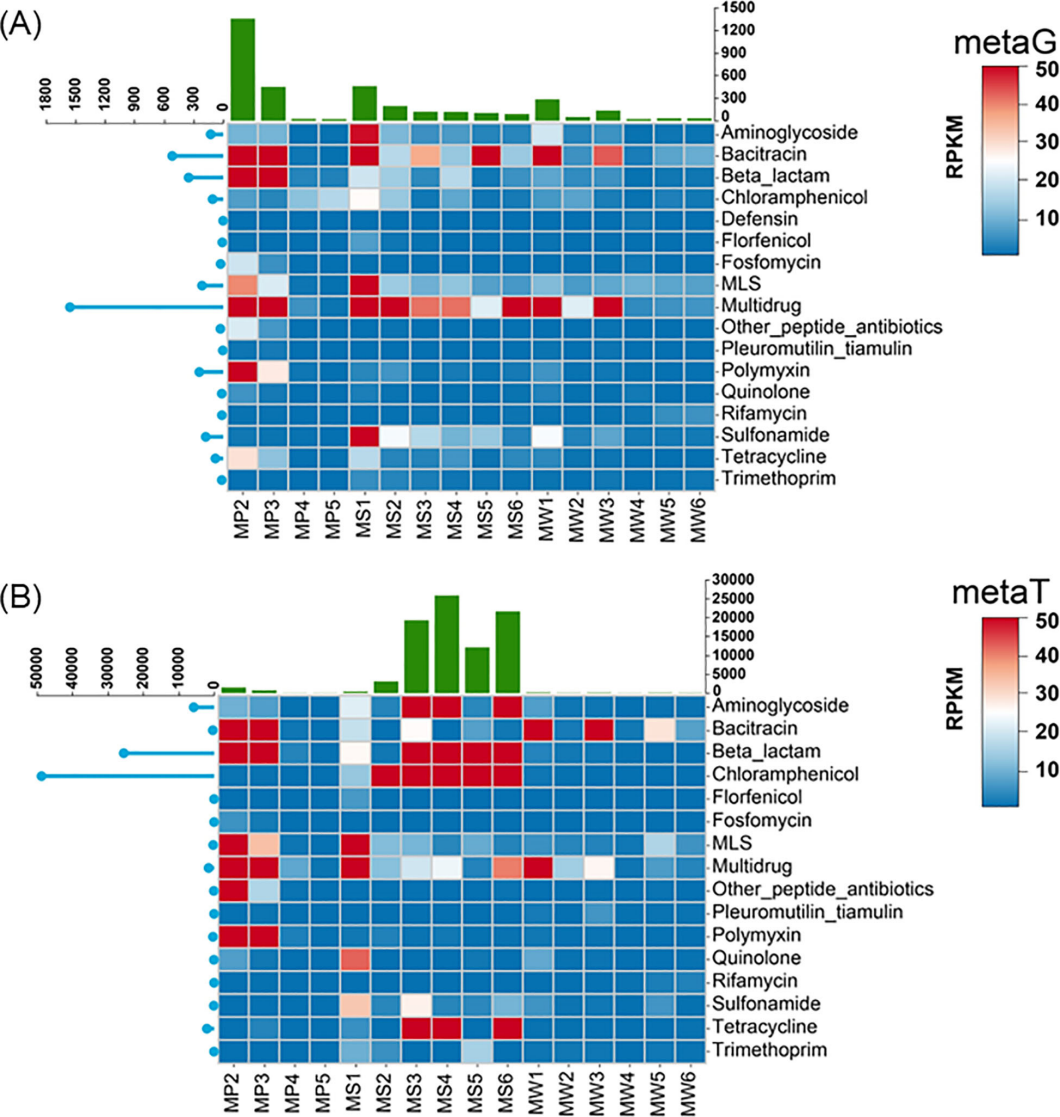
Integrated meta-omics map of ARG abundance **(A)** and expression **(B)**. MP, plant samples; MS, sediment samples; MW, water samples.

Considering that short reads typically come from qualified high-throughput sequencing data, which covers most of the genomic regions in a sample, this helps ensure that even low-abundance genes can be detected, especially in complex environmental samples. Across plants, sediments, and water, cell number estimates derived from metaT were consistently and significantly higher than those from metaG (Wilcoxon *p* < 0.001 for all three media) (**Supplementary Figure S1**). The effect size was greatest in water, intermediate in sediments, and smallest in plants, which indicated a higher proportion of metabolically active microorganisms in water and sediments and a comparatively constrained activity in plant-associated communities. However, the ARG richness identified by metaT was significantly lower than in metaG, demonstrating that metaG could provide broader coverage of the ARGs (**Supplementary Figure S1**). Specifically, **Figure 2** delineates the ARG landscape from both metaG and metaT perspectives, identifying 25 types of ARGs and the three compartments (plant, sediment and water) containing different compositions of ARGs revealed by both metaG and metaT (**Supplementary Figure S2**). In metaG, the plant was dominated by ARGs associated with multidrug and polymyxin. Except in plant-associated samples, the expression of ARGs (in metaT) in the sediment and water was significantly higher than that in metaG, particularly in sediments, where β-lactam and chloramphenicol resistance genes prevailed. In both metaG and metaT, the abundance and expression of ARGs declined markedly in plants, sediments, and water, demonstrating a substantial mitigating effect of the CW on ARGs.

Contig-level assembly and annotation were undertaken to identify and differentiate the genetic carriers of ARGs and to assess their horizontal transfer potential. Assemblies were produced under three strategies, namely single-sample, grouped, and co-assembly, to maximize contig recovery. Relative to co-assembly alone, the single-sample and grouped strategies increased contig counts by 5.2–16.4%. After dereplication across strategies, the nonredundant contig set was about 1.5-fold larger than that obtained from co-assembly alone, providing a higher-coverage reference for downstream ARG and MGE analyses. In total, 1,143 ARG-carrying contigs were confidently identified, spanning 17 type ARGs. In metaG, ARG abundance in the plant was significantly higher than in the water and sediments, being 2.7 times and 7.6 times, respectively. In metaT, sediments were dominant, with ARG expressed 3381.5 times and 1455.1 times higher than in plants and the water, respectively. The mRNA/DNA ratio indicated markedly elevated transcriptional activity in sediments (Ota et al., 2024), with a value of 126.4, compared with 0.71 in the water and 0.77 in the plant. This pattern implied varying degrees of transcriptional silencing in the microbial community associated with plant and water and identifies sediments as a high-activity hotspot for ARGs. Sediments experience prolonged co-selection from slow-release, subinhibitory concentrations of antibiotics and reside within hypoxic biofilm microenvironments, conditions that sustain ARG transcription (Flores-Vargas et al., 2021). By contrast, the water and plant are more susceptible to dilution and short residence times, which hinder the maintenance of persistent selection pressure. Moreover, extracellular DNA and nonviable cells in the rhizosphere and water can inflate DNA-level signals without corresponding mRNA, resulting in high abundance but low activity (Savin et al., 2023).

Regarding ARG removal, the total abundance of ARGs in the water decreased from 276.1 to 30.9 RPKM, with expression decreasing from 303.5 to 19.2 RPKM (**Figure 2**). In the plant, the total abundance of ARGs declined from 1,313.2 to 20.1 RPKM, with expression declining from 1,273.7 to 1.0 RPKM. In the sediment, the total abundance of ARGs decreased from 456.2 to 87.1 RPKM, whereas expression abundance increased from 303.5 to 21,389 RPKM. Plants at the inlet serve as the primary hotspots of ARG abundance and expression, whereas at the outlet, sediments dominate in both abundance and expression of ARGs in CWs. CWs achieved substantial reductions in ARG abundance across the three matrices, with values of 98.5% in plants, 80.9% in sediments, and 88.8% in water, respectively. However, while ARG activity decreased concomitantly in the water and plant, it increased by an enrichment factor of 69.5 in sediments. Overall, CW effectively reduced the abundance and expression of ARGs, thereby mitigating risks in the plant and water. In sediments, however, despite a decline in abundance, transcriptional activity increased markedly, indicating that low abundance does not mean low activity. Accordingly, assessments of transcriptional activity should be incorporated into sediment ARG monitoring.

The type of ARGs in different compartment were further compared by both abundance and expression. Results showed that in metaG, the spectrum of ARG types was dominated by multidrug and bacitracin type across the plant, sediment, and water, whereas sulfonamide type ARGs were commonly detected in the water and sediments. Sulfonamide-type ARGs are tightly linked to class 1 integrons, and multidrug-type ARGs determinants are often carried on conjugative plasmids and transposons that promote co-transfer and co-selection of multiple ARGs (Chen et al., 2016; Cacace et al., 2019). These mobility features help such determinants maintain a competitive abundance advantage within microbial communities. At the transcriptional level (metaT), the ARG type of plant sample consistently exhibited the highest expression of multidrug tolerance–related pathways, consistent with Yan et al. (2021). This is because plant-derived metabolites and rhizosphere conditions can induce bacterial multidrug efflux systems and other stress responses, thereby elevating transcriptional output in plant-associated communities (Tan et al., 2025). The elevated expression of multidrug, β-lactam, and chloramphenicol resistance in the water and sediments is attributable to the extensive clinical and aquaculture use of these agents, together with their persistence and sorption in aquatic and benthic matrices that sustain residual exposures sufficient to induce transcription of the corresponding resistance pathways (Wang et al., 2023). Interestingly, following CW treatment, the activity of multiple ARGs decreased, whereas aminoglycoside-type ARG activity increased markedly.

Collectively, these results indicate that plants act as the primary aggregation hotspot for ARGs at the DNA level but contribute comparatively little transcriptional activity, whereas sediments, despite lower genomic loads, function as the principal activation zone with high transcriptional output. This compartmental decoupling of abundance and activity clarifies risk localization and supports sediment-focused management and transcription-based monitoring in full-scale CWs.

### 3.2 Identification of active ARG carriers in full-scale CWs

**Figure 3:**
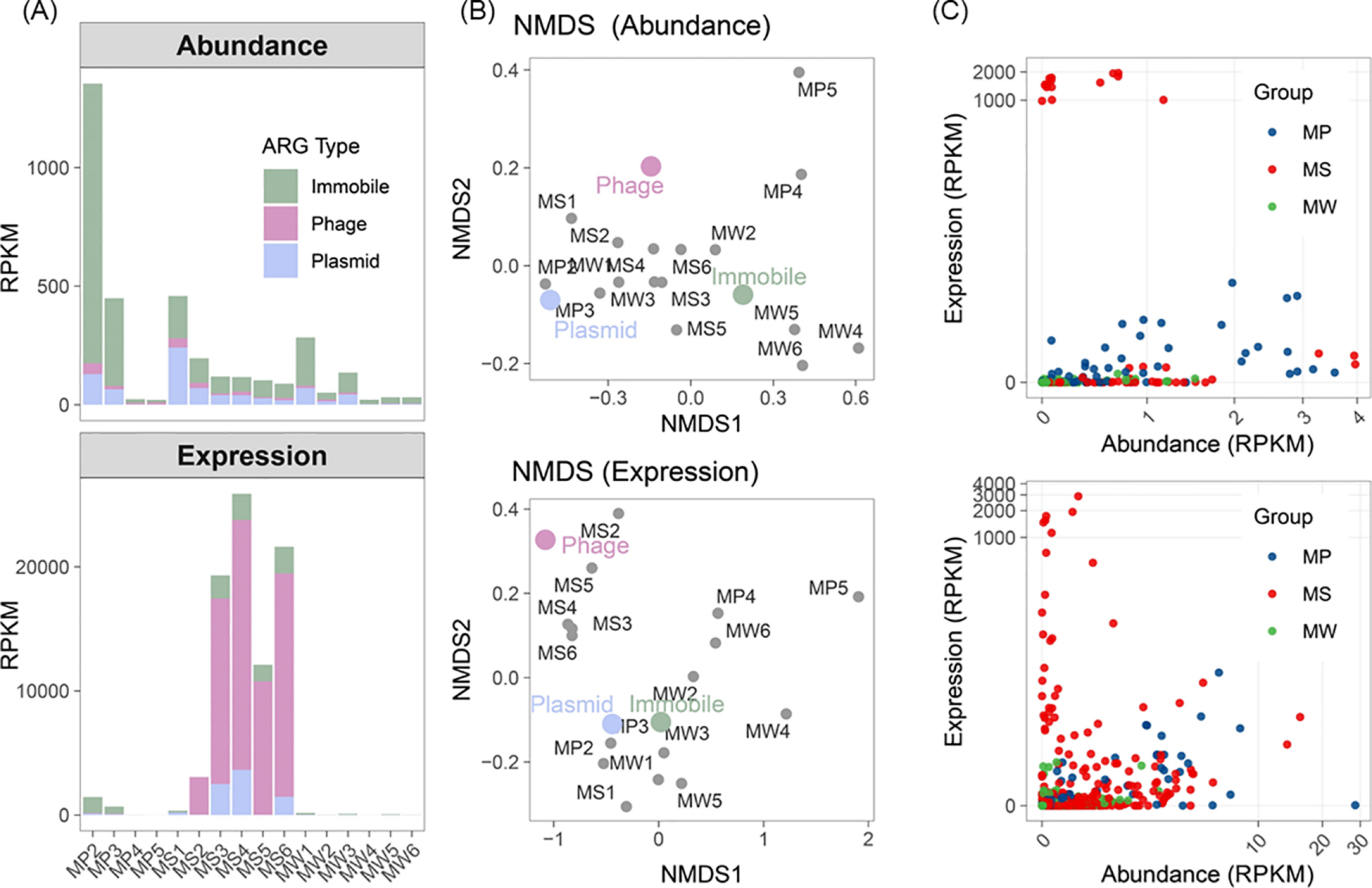
ARG carriers in the CW. **(A)** Classification of ARG carriers in metagenomes (metaG) and metatranscriptomes (metaT) across immobile, plasmid, and phage categories; **(B)** NMDS ordination of samples and ARG carriers; carrier symbols indicate the centroid of their average distribution across samples; and **(C)** Scatterplots comparing metaG and metaT abundances of ARGs located on phages (top) and plasmids (bottom).

To resolve which carriers actively propagate ARGs—particularly within sediments—we combined DL and alignment-based approaches to classify ARG-carrying contigs as plasmid or phage and to refine prophage boundaries (**Figure 1B**). The DL models (PhaBOX2 and PLASMe) captured more phages and plasmids, while homology/marker searches provided conservative validation; scores from both streams were integrated to yield high-confidence carrier calls (**Supplementary Figure S3**). In total, 186 plasmid-derived, 56 phage-derived, and 901 putative non-mobile elements were identified in all the ARG-carrying contigs. We then mapped metaG and metaT reads to these MGE backbones to compare the abundance and expression of carried ARGs (**Figure 3A**). In metaG, plasmids dominated as the principal ARG carriers, with abundances 3.7 times those of phages. In terms of spatial distribution, plasmid-derived ARGs peaked in sediments, were intermediate in plants, and were lowest in the water column. With respect to ARG type composition, plasmids harbored 13 types of ARGs and phages harbored 7 types of ARGs. Consistent with the co-occurrence networks (**Supplementary Figure S4**), plasmid-associated ARGs formed the broadest backbone and dominated node degree and inter-class links across matrices, whereas phage-derived links were narrowly distributed around core classes and were organized as discrete modules (**Supplementary Table S5**). Specifically, plants exhibited plasmid backbones anchored in MLS, polymyxin, and trimethoprim; sediments showed plasmid connectivity concentrated on tetracycline and aminoglycoside, with additional connections to β-lactam and multidrug and with higher modularity and fewer bridges between modules; water showed a β-lactam to quinolone axis of cross-class connectivity. Phage-driven ARGs showed medium-specific dominance patterns, with multidrug and polymyxin-type ARGs prevailing in plant samples, multidrug-, MLS-, and tetracycline-related ARGs dominating sediments, and quinolone- and multidrug-type ARGs being most prominent in the water. Following CW’s treatment, plasmid-derived ARGs decreased by 99.4% in plants, 92.4% in sediments, and 94.6% in the water, whereas phage-derived ARGs decreased by 79.6%, 75.3%, and 87.1%, respectively.

At the transcriptomic level, ARG transcription in water and plant samples predominantly mapped to plasmids, and no phage-associated ARG transcripts were detected in the water. However, in sediments, ARG transcription predominantly mapped to phages (**Figure 3B**), with phage-associated ARG expression 276.4-fold higher than plasmid-associated expression. The expressed plasmid-derived ARGs were chiefly from the aminoglycoside and tetracycline type ARG, while the expressed phage-associated ARGs were primarily β-lactam, chloramphenicol, and trimethoprim. Specifically, these three types of ARGs are contained in each phage contig, which indicates the co-infectious ability (**Supplementary Figure S5**). Following CW’s treatment, plasmid-derived ARGs decreased by 99.9% in plants and 92.4% in the water, while phage-derived ARGs in plants declined by 99.2%. Interestingly, in sediments, the expression of both plasmid- and phage-derived ARGs was enriched. The plasmid increased by 6.2-fold, whereas the phage increased by 2,112.3-fold. The proportion of phage-derived ARGs in the total ARG transcripts increased from 4.0% in the influent to 92.5% in the effluent.

Abundance-expression scatter plots (**Figure 3C**) further demonstrated that both phage and plasmid-derived ARGs in the sediment exhibited a “low abundance, high expression” cluster. Prolonged and widespread clinical and environmental antibiotic use elevated the baseline prevalence of β-lactam (e.g., *blaTEM/blaCTX-M*) and chloramphenicol (*cat*) resistance genes in bacterial communities, making them more readily captured and carried by phages during induction or transduction (Zhang et al., 2019). Based on network metrics and visualization, transcriptional network complexity decreased in the order plants > water > sediments (**Supplementary Figure S4**; **Supplementary Table S5**). Plants exhibited higher node and edge counts, greater network density, and a larger proportion of inter-class links; water was intermediate; sediments were comparatively compact. Regarding carrier contributions, the transcriptional backbones in plants and water were primarily supported by plasmid-derived edges, with plasmids bridging modules and supporting most high-weight connections, whereas the increasing complexity in sediments was driven mainly by phage-derived edges, indicating plasmid-led broad transcriptional connectivity in plants and water and phage-amplified coupling among key ARG classes in sediments.

Our results denoted that ARGs exhibited clear compartmentalization: plants were dominated by DNA-level enrichment, whereas sediments were transcriptionally dominant, marked by a pronounced elevation and expanded share of phage-linked expression. This spatial pattern aligns with the biofilm-niche paradigm in which high cell density and surface attachment render sediments and attached habitats hotspots for HGT, supporting the maintenance and mobilization of MGEs—particularly plasmids and phages (De La Cruz Barron et al., 2018). At the carrier level, plasmids showed greater class diversity and a cross-compartment “resident” signature, consistent with field evidence for enrichment of conjugative plasmids in riverine/sediment matrices and with the overall capacity of CW to reduce total ARGs via sorption by substrates, plant uptake, and microbial processing (Gonzalez-Plaza et al., 2019). In contrast, although phages were less abundant genomically, they exhibited disproportionately high expression and an increasing contribution in sediments, indicating a growing functional weight of the phage-mediated pathway. This contrast likely reflects fundamental differences in transcriptional regulation and life-cycle strategies between phages and plasmids: phage-driven ARGs can be co-transcribed during prophage induction, resulting in burst-like transcriptional amplification, whereas plasmid-borne ARG expression is largely governed by host regulatory control and tends to remain comparatively stable in the absence of strong selective pressure (San Millan and Maclean, 2017; Liao et al., 2024). In sediments, biofilm-associated growth, high microbial densities, and pronounced physicochemical gradients may promote host stress responses, thereby facilitating prophage induction and enhanced viral transcriptional activity, which could amplify the relative contribution of phage-driven ARG expression (Merges et al., 2023). This agrees with observations in wastewater-treatment systems that the phage fraction is more persistent, can traverse treatment units, and retains a downstream dissemination potential, underscoring that transduction should not be overlooked in engineered systems (Ji et al., 2025). Overall, CW substantially reduced the genomic load of ARGs, but the “de-abundance” versus “de-activity” responses diverged by carrier: relative to plasmids, phage-associated signals often showed higher residuals and greater transmissibility post-treatment, a pattern repeatedly reported across diverse wastewater contexts (Calero-Caceres and Muniesa, 2016). Taken together, while CWs lower the total ARG burden, the dominant contributors to system residuals and off-site risk increasingly point to phage-derived ARGs in sediments.

### 3.3 Pathogenic hosts of ARG-carrying phages: distribution and transcriptional activity

**Figure 4:**
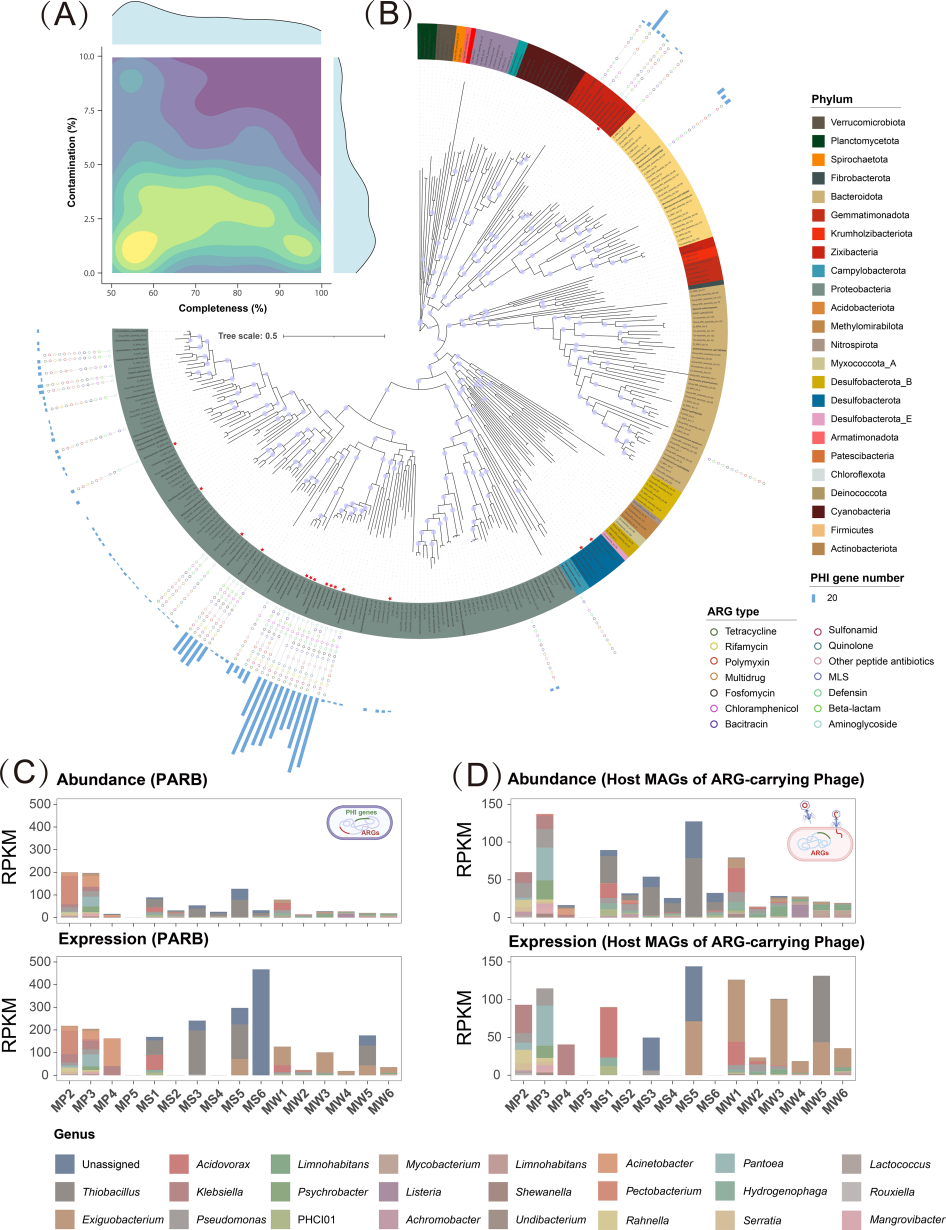
Identification and dynamics of pathogenic antibiotic-resistant bacteria (PARB) and host MAGs of ARG-carrying phage. **(A)** MAG quality statistic, including genome completeness and contamination; **(B)** Phylogenetic tree of recovered MAGs; **(C)** Abundance and transcriptional activity of PARB across compartments; and **(D)** Abundance and expression of host MAGs of ARG-carrying phages.

Consistent with the central dogma, transcription of phage-borne ARGs requires active infection of a bacterial host. To identify these hosts, we used the deep-learning classifier CHERRY, which links phages to bacteria by integrating compositional and coverage co-variation with CRISPR spacer matches. Candidate hosts were derived from metagenomic binning, enabling phage–host assignments on a genome-resolved basis. Using a unified binning workflow on metagenomic datasets, 310 nonredundant metagenomic assembled genomes (MAGs) were recovered (completeness >50%, contamination <10%, **Figure 4A**). A total of 16 phage hosts were identified from MAG assignments, spanning the phyla Desulfobacterota, Firmicutes, and Proteobacteria and encompassing 12 genera, including *Pantoea*, *Klebsiella*, and *Pseudomonas* (**Figure 4B**). At the host level, *Klebsiella* and *Pantoea* emerged as the most prominent carriers: the former was most strongly connected to polymyxin and multidrug, whereas the latter was primarily associated with multidrug, suggesting functional partitioning and preference among ecological guilds with respect to ARG type (Zhang et al., 2023; Di Cesare et al., 2024). 11 of the 16 phage hosts carried both ARGs and pathogenic factors (PHI genes) and were designated as potentially pathogenic antibiotic-resistant bacteria (PARB). As shown in **Figures 4C, D**, in plants, sediments, and water, pathogenic phage hosts made up 80.7%, 55.3%, and 30.4% of total host abundance, respectively; their corresponding expression proportions were 98.2%, 72.2%, and 34.5%. In total, 37 MAGs identified as PARB. Proteobacteria was the dominant phylum, contributing more than 90% of cumulative abundance and expression. At the genomic level, the plants contained 31 PARB (dominant genera: *Pectobacterium*, *Pantoea*, *Pseudomonas*), sediments contained 14 PARB (dominant: *Thiobacillus*), and the water column contained 25 PARB (dominant: *Limnohabitans*, *Exiguobacterium*). Overall PARB richness and abundance were highest in plants, indicating this compartment acts as a PARB enrichment hotspot. At the transcriptomic level, detectable expression was observed for 29 PARB in plants (dominant expressed genera: *Pectobacterium*, *Klebsiella*, *Acinetobacter*) and for 8 PARB in sediments (dominant: *Thiobacillus*). After CWs treatment, PARB in the water decreased by 63.5% in genomic abundance and by 80.1% in expression. PARB in plants were reduced to undetectable levels in both abundance and activity. PARB in sediments decreased by 63.5% in genomic abundance but increased 183.4% in expression.

CWs showed substantial overlap between PARB and phage hosts. Pathogenic phage hosts were enriched and highly active in plant and sediment, contributing to PARB abundance and expression. Together, these patterns indicated an active phage-mediated pathway that elevated the risk of ARG transfer (via transduction) within and among pathogenic. This is largely attributable to the fact that the genomes of PARB are often enriched in functional prophages carrying virulence factors, ARGs, and auxiliary metabolic genes (AMG) (Gamaliel. et al., 2022). Such genetic cargo substantially enhances the virulence and survival of pathogenic bacteria within the host, thereby promoting the long-term maintenance of prophages in pathogenic populations (Liao et al., 2024). At the same time, upregulation of expression occurred in low abundance of phage hosts in sediments and plants, suggesting that the microenvironment can promote the interaction between phages and pathogenic hosts. Previous studies have shown that phage dynamics were highly sensitive to microbial density, and the high density and long residence microenvironment provided by biofilms could increase the efficiency of phage transmission to hosts (Liu et al., 2023; Xia et al., 2025). Phage–plasmids are frequently detected in wastewater treatment systems. These elements are both infectious and capable of stable replication, and they can carry and disseminate ARGs, thereby increasing the potential for pathogenic bacteria to develop antibiotic resistance (He et al., 2022; Shan et al., 2023). Dense plant-associated biofilms and root-exudate–driven quorum sensing jointly regulate phage adsorption and replication, thereby increasing the likelihood of infection initiation and enhancing host–phage interactions (Leon-Felix and Villicana, 2021). In summary, phages provided a viable pathway for ARGs to enter and disseminate within and among pathogenic populations through overlap with PARB host ranges in CWs. This pathway was more readily induced in high-density, biofilm-associated niches (e.g., sediments), thereby amplifying ecological risk.

### 3.4 Quantifying the cross-compartment distribution of pathogenic hosts of ARG-carrying phages in the CW

**Figure 5:**
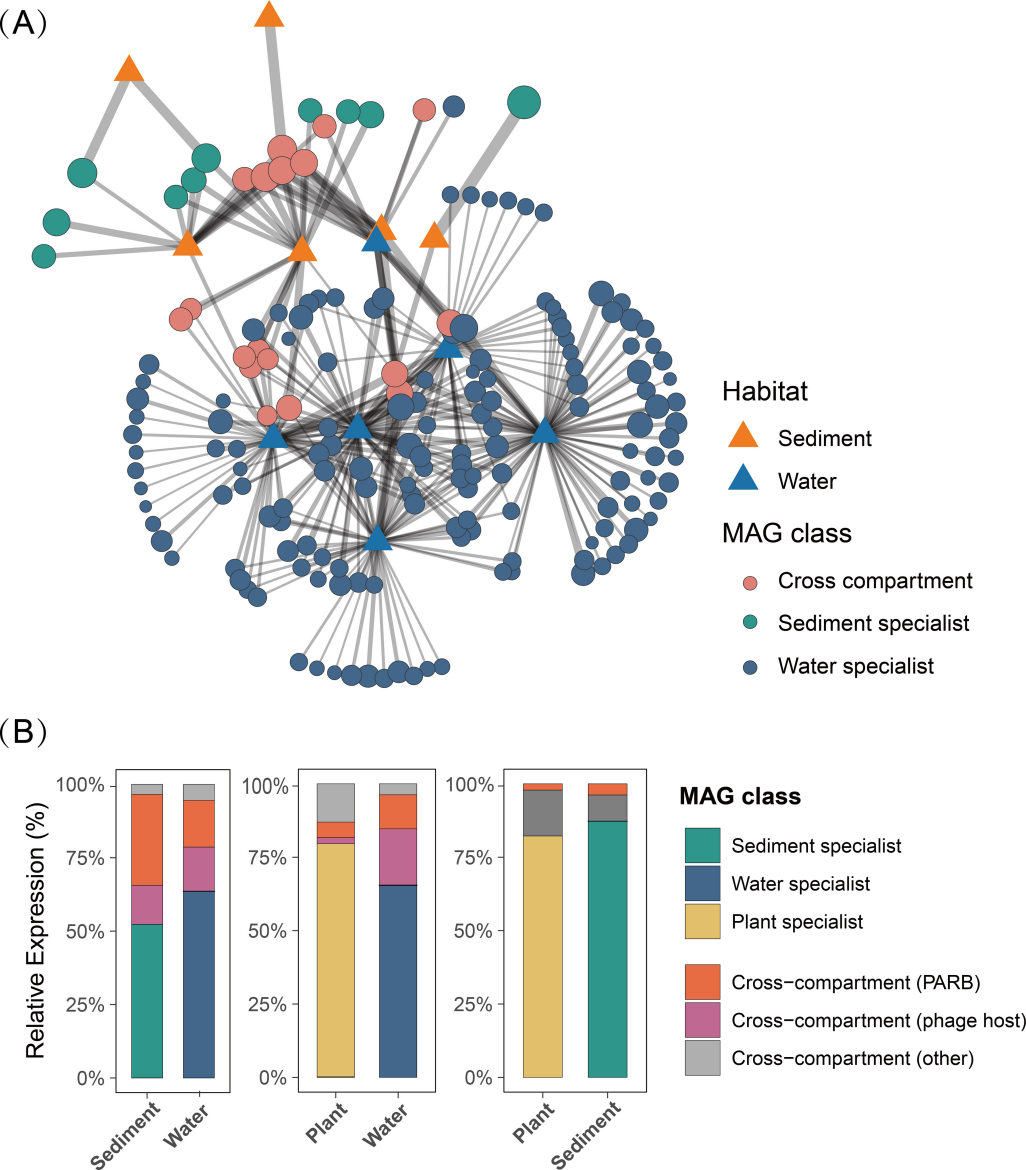
Cross-compartment distribution of pathogenic hosts of ARG-carrying phages in the CW. **(A)** Bipartite network linking water and sediment samples to cross-compartment MAGs. Sample nodes are shown as habitat-specific symbols, whereas MAG nodes are depicted as filled circles whose color denotes MAG class and whose size reflects the log-transformed mean RPKM across all samples. Edge widths are proportional to the log-transformed RPKM values, emphasizing stronger associations. Node positions were determined using the stress layout algorithm; and **(B)** Relative transcriptional contribution of different MAG categories to the total community activity across compartments.

MAGs with cross-compartment transmission potential and their transcriptional contributions were further quantified using a bipartite network framework that explicitly links individual MAGs to their water, sediment, and plant-associated habitats. Within this framework, the cross-compartment transmission potential of ARG-carrying phage hosts and PARB-encoding MAGs was specifically analyzed. By quantifying pairwise transmission potential between habitat pairs, it was found that the water–sediment interface represents a major hotspot for both PARB-encoding and phage-host MAGs, indicating particularly tight connectivity between these two compartments. As shown in **Figure 5A**, 18 MAGs remained actively expressed across the sediment–water compartments, underscoring their prominent role in mediating cross-habitat connectivity. Together, these cross-compartment MAGs accounted for 47.6% of the total transcriptional activity in sediments and approximately 38.92% in the overlying water (**Figure 5B**). Within this group, cross-compartment phage-host MAGs and PARB-encoding MAGs contributed 18.9% and 20.3% of the sediment transcription, respectively, indicating that a substantial fraction of the active sediment community is both phage-associated and potentially mobile. By contrast, only five cross-compartment MAGs were detected in the plant–sediment assemblage, and their transcriptional contributions to both compartments were minor (**Supplementary Figure S6**). In the water–plant assemblage, 16 cross-compartment MAGs were identified, but they contributed only ~10.6% of total transcription in the water and ~15.5% in the plant-associated fraction (**Figure 5B**). Collectively, these patterns indicate that a disproportionately large share of functionally active microbial populations with phage-host and PARB signatures are capable of moving between sediments and overlying water while maintaining high transcriptional activity in both habitats. This finding is consistent with the previously observed similarity in the distribution of phage-host and PARB categories between sediments and water, highlighting the sediment–water interface as a critical hotspot for the cross-compartment circulation of ARG-associated microbial hosts. Taken together with the higher abundance of bacteriophages carrying ARGs detected (**Figure 3**), these findings suggest that sediments, as a key environment for phage hosting and PARB enrichment, represent an important reservoir for the accumulation and environmental dissemination of ARGs, and that their associated risks should not be overlooked.

Environmental factors are generally regarded as key drivers of phage-mediated ARG migration and PARB transfer across different compartments in aquatic environments (Li et al., 2024). To this end, we performed environmental correlation analyses to disentangle the relationships between phage-mediated ARG dynamics and key physicochemical gradients, aiming to pinpoint the factors that may actively promote phage activity and ARG transfer across habitats. Mantel test results revealed positive correlations between phage-driven ARG profiles and sediment ORP, EC, as well as lomefloxacin concentrations (*p* < 0.05; **Supplementary Figure S7**), suggesting that redox conditions, ionic strength, and antibiotic residues collectively shape phage-associated ARG dynamics in sediments. Changes in redox conditions have been reported to activate latent MGEs, thereby promoting the recombination and dissemination of ARGs within microbial communities (Han et al., 2023). This mechanism can significantly alter the ARG spectrum without causing substantial changes in the overall community structure.

In sediment and aquatic environments, ORP has been shown to be significantly associated with ARG abundance as well as viral/bacteriophage community structure, which indicates that ARGs and the environmental virome are jointly regulated by redox gradients (Liu et al., 2025). In general, low ORP values (i.e. stronger reducing conditions) in sediments are typically associated with high organic matter content, active reduction of sulfate and metal oxides, elevated bacterial densities and varying degrees of nutrient limitation (Hoshino et al., 2020). Such conditions are thought to favor lysogenic lifestyles and the long-term maintenance of prophages within host genomes, leading to an accumulation of prophage reservoirs in reduced sediments (Fischer et al., 2003). Under these circumstances, prophages carrying ARGs or AMGs are more likely to be retained within host populations under antibiotic pressure (Liao et al., 2024). Phage AMGs frequently complement or enhance host metabolic outputs, thereby facilitating host survival under polluted or otherwise stressful environmental conditions and promoting the long-term, quasi-mutualistic co-existence between phage and their hosts (Sun et al., 2023). Over longer time scales, these redox conditions may influence host activity and phage life cycles, thereby indirectly amplifying the dissemination potential of phage-drived ARGs. EC serves as an integrated indicator of dissolved salts and co-pollutants in sediment pore water, reflecting not only salinity and ionic strength but also the intensity of external inputs from wastewater and agricultural non-point sources (Akinnigbagbe et al., 2025). Numerous studies have shown that EC is one of the key factors regulating the spatial distribution of ARGs in soils and aquatic environments (Zhang et al., 2025a). Regions with elevated EC often exhibit higher loads of nutrients, metals, and organic pollutants, which through co-selection and community filtering, markedly promote the enrichment and diversification of ARGs. High ionic strength may also facilitate biofilm formation and the development of high-density microbial aggregates, thereby increasing the frequency of contact between bacteriophages and their hosts and enhancing the likelihood of phage-mediated HGT. On the other hand, elevated EC also reshapes the physicochemical context in which phage–host interactions occur. Specifically, higher EC is typically accompanied by increased concentrations of inorganic ions, which help to screen electrostatic repulsion between phage capsids and bacterial cell surfaces, enhance phage adsorption efficiency, and promote phage attachment and retention on mineral and organic particles, thereby creating local phage-enriched microenvironments within sediment pores and biofilms (Armanious et al., 2016).

In addition, Mantel test results indicated a positive correlation between lomefloxacin (LM) and phage-mediated ARGs. As a fluoroquinolone antibiotic, LM and related compounds are relatively persistent in water and sediments and can substantially alter microbial community structure and exert strong selective pressure on quinolone resistance genes, thereby promoting the enrichment of associated ARGs (Zou et al., 2024). At the same time, quinolones have been widely shown to induce the bacterial SOS response and trigger the transition of prophages from the lysogenic to the lytic cycle, which enhances phage replication and transduction and increases the frequency of phage-borne ARG transfer (Baharoglu and Mazel, 2014). Taken together, the physicochemical gradients represented by ORP and EC, in combination with residual lomefloxacin and other antibiotics, create a “high-risk” environmental pool in sediments that favors phage–host interactions, MGE activation, and ARG accumulation. Through material exchange across the sediment–water interface, this pool provides a sustained driving force for the export of phage-associated ARGs to the overlying water column, thereby reinforcing the critical role of the sediment–water axis in ARG dissemination in this agricultural aquatic system.

The results of this study highlight important implications for ARG risk monitoring and management in constructed wetlands. Although CWs effectively reduced the overall genomic abundance of ARGs, our activity-resolved analyses revealed that sediments can retain a disproportionate share of transcriptionally active, phage-associated ARGs, representing a residual but potentially high-risk pathway for resistance dissemination. These findings suggest that monitoring strategies based solely on DNA-level abundance may underestimate terminal risks in CW systems. From a management perspective, ARG surveillance in CWs should therefore move toward an activity-aware and carrier-resolved framework. In particular, sediment compartments should be prioritized for routine monitoring, with emphasis on metatranscriptomic indicators of ARG expression and phage-mediated transduction potential. Integrating transcript-based metrics, phage-associated ARG signatures, and host-resolved information could enable earlier detection of high-risk residuals and improve the sensitivity of risk assessment beyond conventional abundance-based approaches. Such targeted monitoring would support more informed management decisions and help ensure the long-term effectiveness of CWs as a barrier against ARG dissemination to receiving waters.

## 4 Conclusion

Using coordinated metaG–metaT profiling and deep-learning–based carrier identification in a full-scale CWs, the ARG dissemination landscape was reconstructed from a carrier-centric perspective. Overall, the CWs substantially reduce both genomic abundance and transcriptional activity of ARGs, yet effluent-end sediments retain residual activity and transmission potential of phage-associated ARGs. These ARGs constitute a high-risk “low-abundance, high-activity” signature in sediment. Furthermore, host resolution based on MAGs identified a Proteobacteria-dominated host of ARG-carrying phages that strongly overlaps with PARB, is enriched and more active in sediments, and indicates that transduction within high-density, biofilm-associated niches is a key source of terminal residual risk. Through cross-compartment transmission along the sediment–water interface, these phage-associated and PARB populations continuously seed the overlying water. Accordingly, ARG risk assessment should shift from static abundance to activity-aware, carrier- and host-resolved evaluation, prioritizing sediment-targeted transcript monitoring and phage/transduction early warning. This framework provides a generalizable technical pathway and decision basis for ARG risk mitigation in constructed wetlands and broader engineered and natural systems. While deep learning approaches have significantly enhanced the ability to analyze MGEs and their hosts, the bioinformatics predictions derived from these methods still lack robust validation through experimental techniques such as Hi-C. Currently, the high transcriptional activity of phage-associated ARGs is used as a proxy indicator of transduction risk. Future research urgently needs to combine long-read sequencing with time-series sampling to more deeply reveal the processes and mechanisms underlying the spread of antibiotic resistance in these systems across both temporal and spatial scales.

## Data Availability

The datasets presented in this study can be found in online repositories. The names of the repository/repositories and accession number(s) can be found in the article/**Supplementary Material**.
