# Supplementary material for: Unraveling the activity of phage-carrying antibiotic resistance genes in constructed wetlands
Source: Front Cell Infect Microbiol. 2026 Feb 9;16:1764958. doi: 10.3389/fcimb.2026.1764958 (PMC12926485; doi:10.3389/fcimb.2026.1764958)
Supplement: Supplementary file 1 [file Table1.docx]

Supplementary Material

## Supplementary Figures


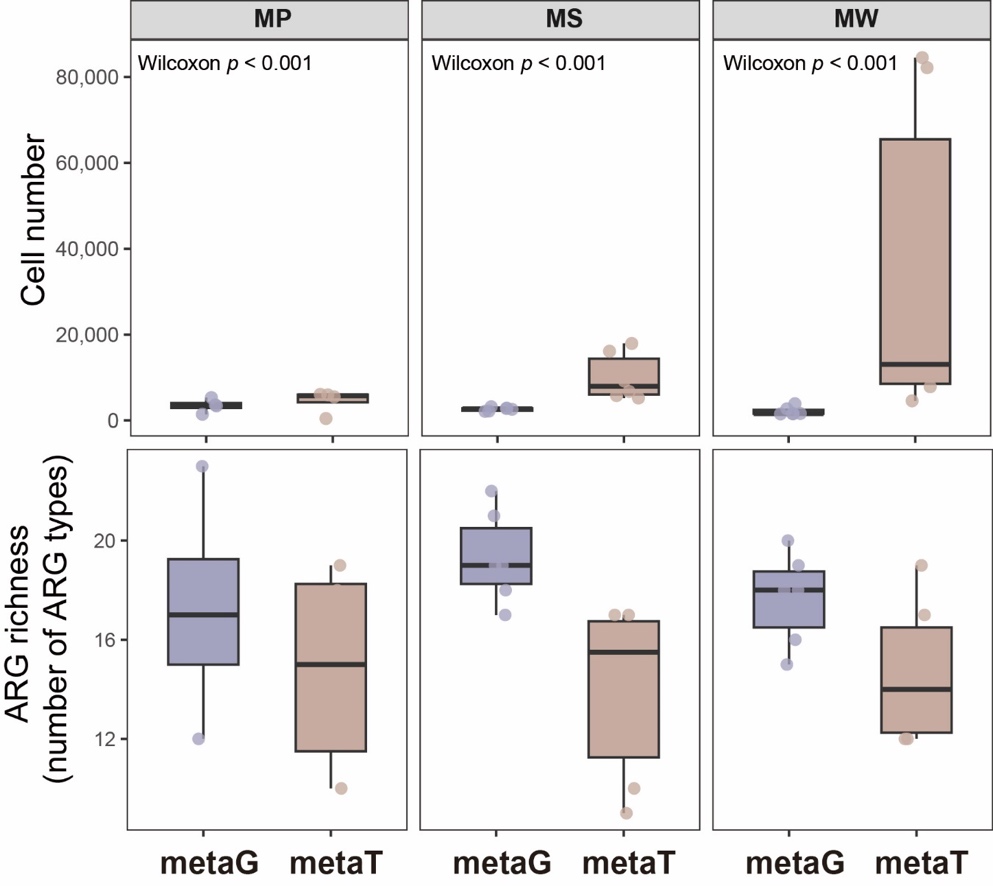


**Supplementary Figure S1.** The cell number and ARG richness identified by metaG and metaT across compartments.


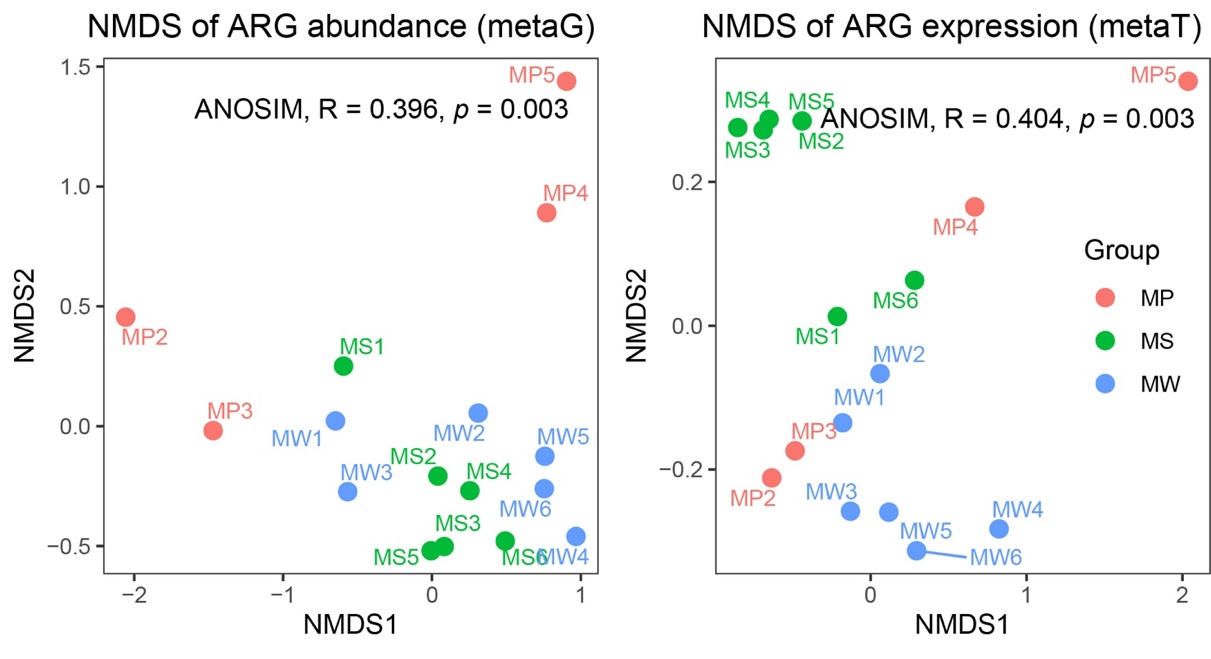


**Supplementary Figure S2.** NMDS ordination of ARG profiling across samples based on short reads.


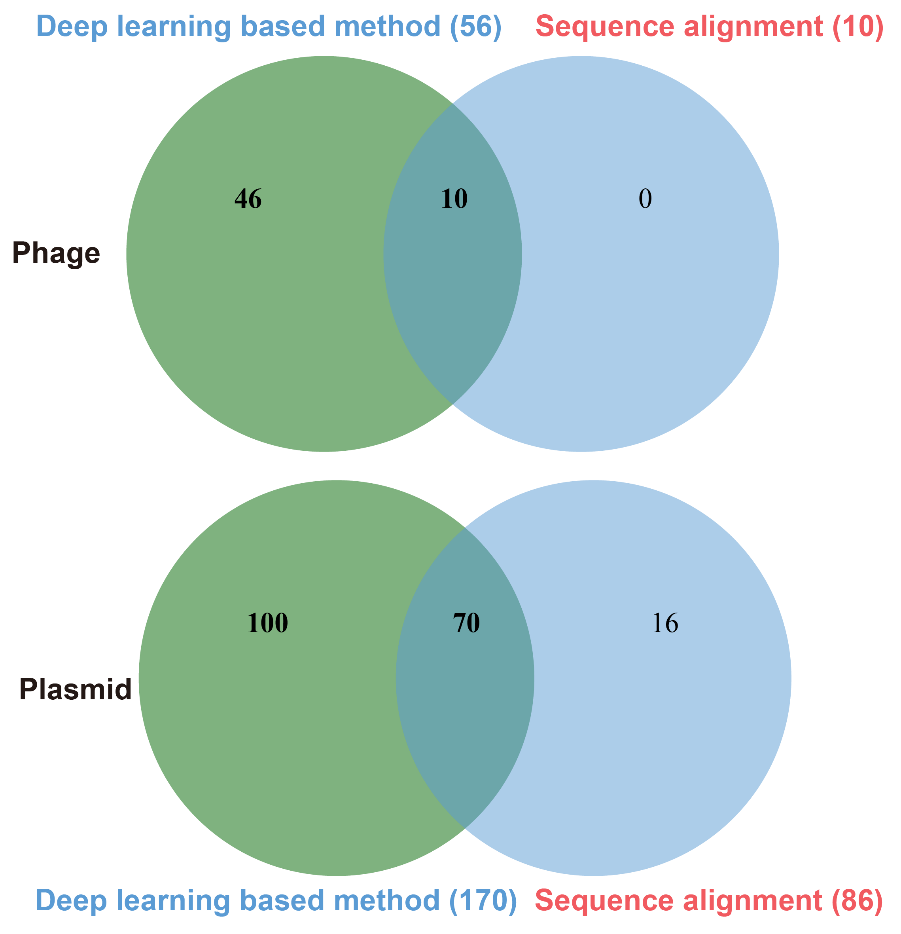


**Supplementary Figure S3.** Results of phage/plasmid identification compare between deep learning and sequence alignment


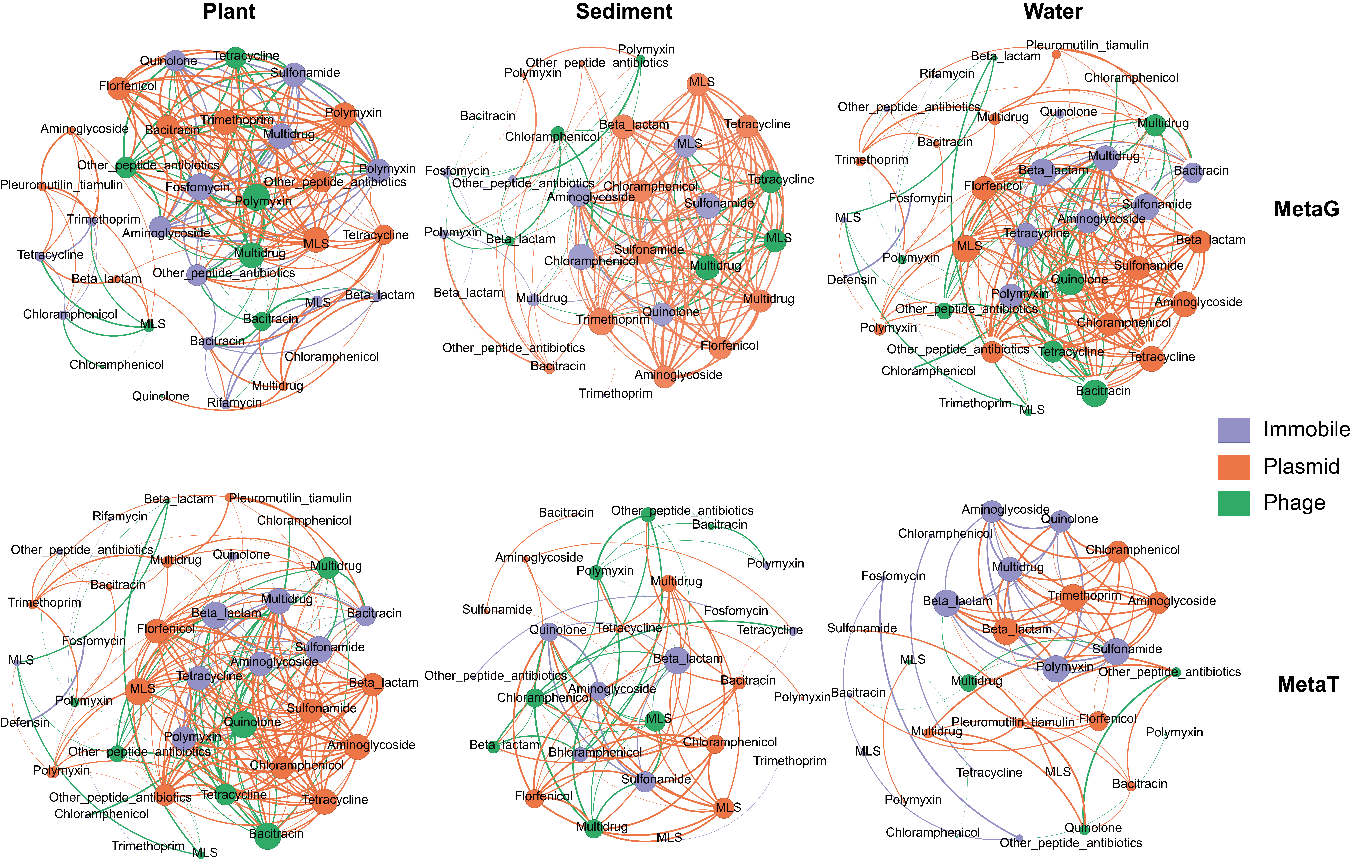


**Supplementary Figure S4.** Collinearity networks of ARG classes across plant, sediment and water (top: metaG; bottom: metaT). Nodes represent ARG type; edges indicate significant co-occurrence; nodes colors denote carrier categories (immobile, plasmid, phage).


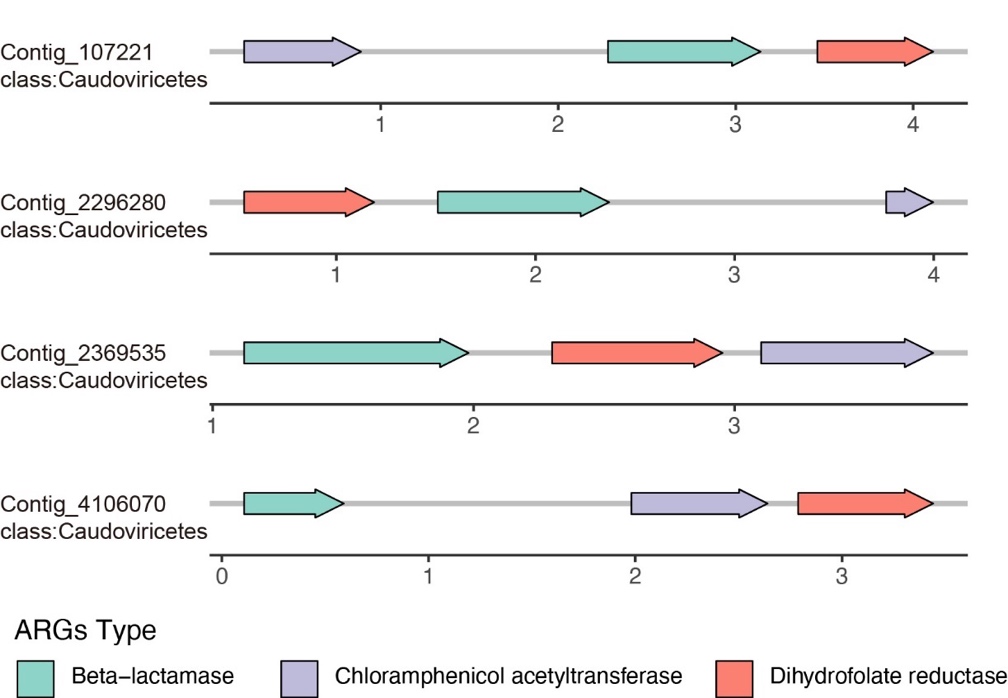


**Supplementary** **Figure S5**. Genomic positions of ARGs on phage contigs.


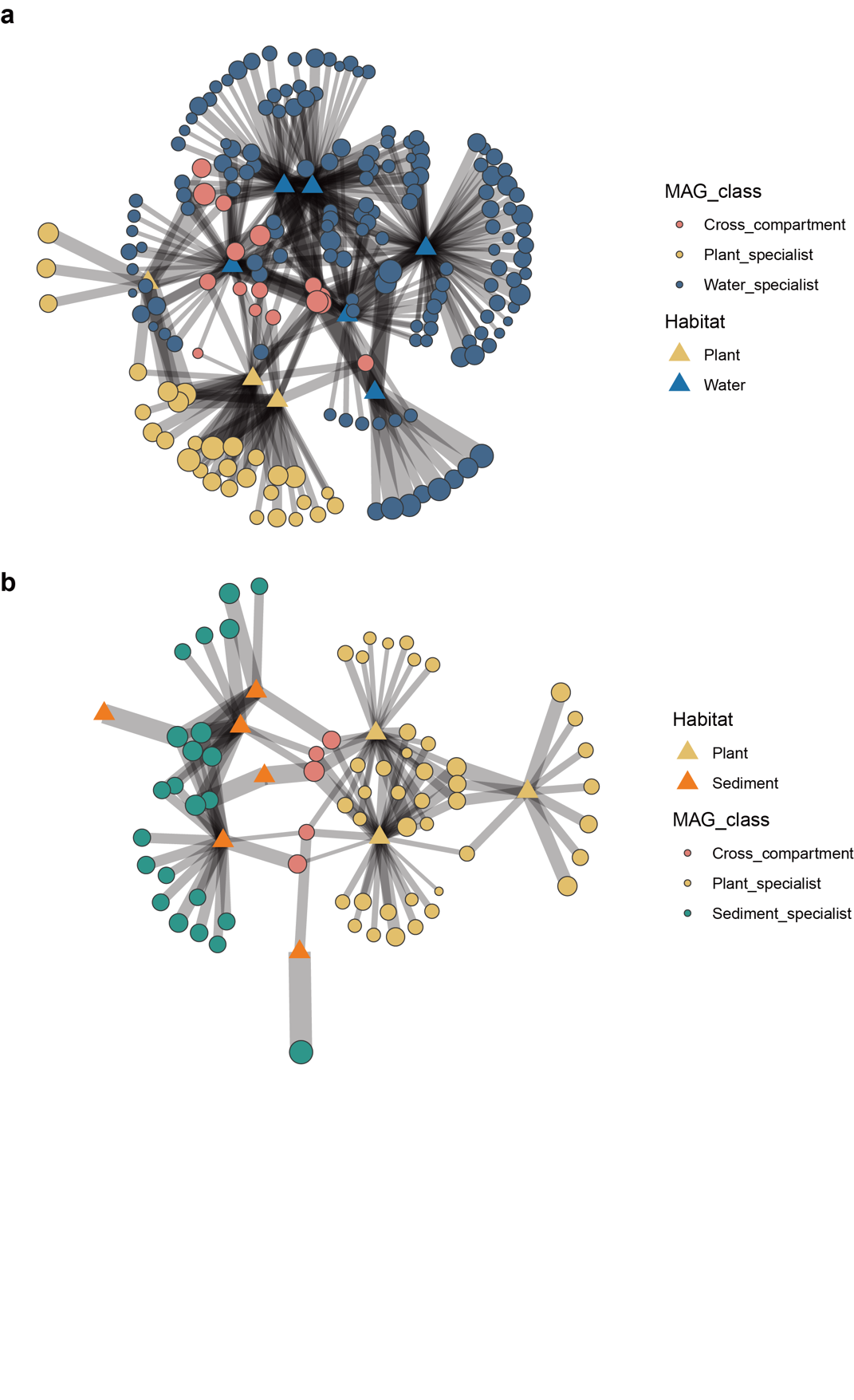


**Supplementary Figure S6.** Cross-compartment distribution of pathogenic hosts of ARG-carrying phages in the plant-water (a) and plant-sediment (b) compartments.


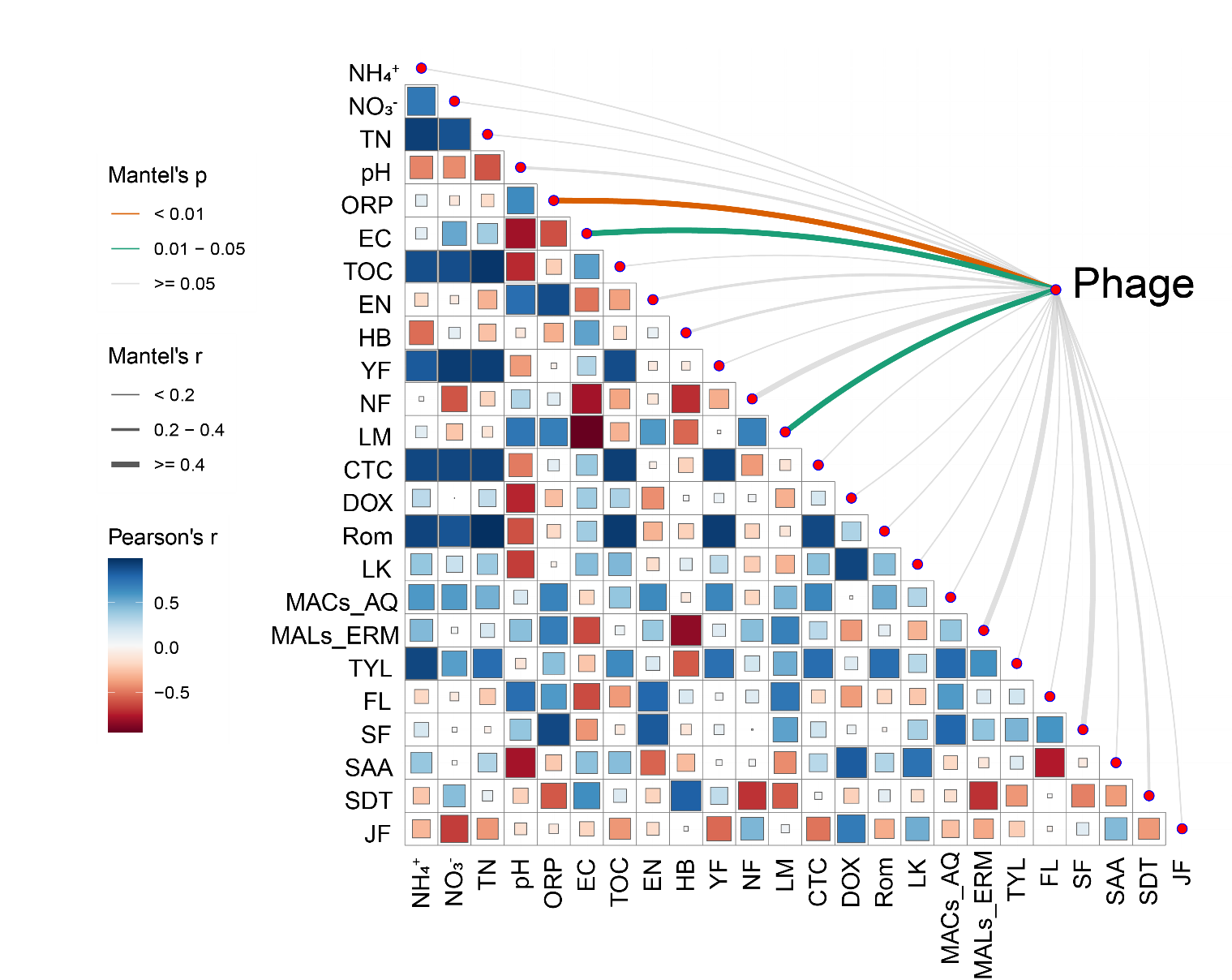


**Supplementary Figure S7.** Pairwise comparisons of environmental factors are shown, with a color gradient denoting Spearman’s correlation coefficient. ARG-carrying phages expression was related to each environmental factor by partial Mantel tests. Edge width corresponds to the Mantel’s r statistic for the corresponding distance correlations, and edge color denotes the statistical significance based on 9,999 permutations..

**Supplementary Tables.**

**Table S1** Sample Sequencing Data Evaluation Statistics in metaG

| Sample sites | Raw data base  (bp) | Number of Reads | GC  (%) | Q20  (%) | Q30  (%) |
| --- | --- | --- | --- | --- | --- |
| MW1 | 8341756990 | 752213 | 54.68 | 94.83 | 84.81 |
| MW2 | 8766748184 | 471145 | 44.44 | 96.46 | 89.36 |
| MW3 | 10517635296 | 114935 | 51.87 | 97.09 | 91.22 |
| MW4 | 15910689369 | 181398 | 41.4 | 96.71 | 89.99 |
| MW5 | 11123786229 | 121804 | 46.15 | 96.62 | 89.84 |
| MW6 | 9555393927 | 105323 | 46.03 | 96.28 | 88.8 |
| MS1 | 11030349457 | 318498 | 57.79 | 95.96 | 88.01 |
| MS2 | 10650862246 | 308646 | 57.8 | 96.01 | 88.14 |
| MS3 | 7624458164 | 15851 | 61.28 | 97.28 | 91.32 |
| MS4 | 10124819803 | 296860 | 58.85 | 97.16 | 91.26 |
| MS5 | 11513788769 | 129119 | 58.3 | 97 | 91.07 |
| MS6 | 9189405343 | 146707 | 60.32 | 96.71 | 89.79 |
| MP2 | 13144152161 | 381168 | 52.13 | 96.53 | 89.53 |
| MP3 | 11315460608 | 127581 | 44.66 | 97.52 | 92.36 |
| MP4 | 8883294515 | 98430 | 45.14 | 96.75 | 90.18 |
| MP5 | 10358160849 | 115130 | 41.56 | 97.78 | 93.21 |

**Table S2** Sample Sequencing Data Evaluation Statistics in metaT

| Sample sites | Raw data base  (bp) | Number of Reads | GC  (%) | Q20  (%) | Q30  (%) |
| --- | --- | --- | --- | --- | --- |
| MW1 | 19442056010 | 221455 | 53.92 | 97.6 | 92.9 |
| MW2 | 10174273399 | 94119 | 47.71 | 96.82 | 90.74 |
| MW3 | 20379984238 | 215419 | 50.76 | 97.31 | 92.16 |
| MW4 | 24482720696 | 251659 | 49.02 | 97.03 | 91.37 |
| MW5 | 9017942620 | 75298 | 50.82 | 98 | 93.82 |
| MW6 | 17302731846 | 41160 | 47.65 | 97.14 | 91.93 |
| MS1 | 12597549347 | 144036 | 50.27 | 97.58 | 92.74 |
| MS2 | 9302646896 | 105348 | 47.11 | 97.72 | 93.14 |
| MS3 | 9375768130 | 32564 | 52.96 | 96.07 | 91.44 |
| MS4 | 9985534052 | 21154 | 52.65 | 95.71 | 91.57 |
| MS5 | 8323727822 | 82477 | 50.59 | 97.34 | 92.66 |
| MS6 | 11407571366 | 20113 | 52.76 | 95.69 | 91.57 |
| MP2 | 17300514614 | 191446 | 51.29 | 97.26 | 92 |
| MP3 | 28299801698 | 310822 | 53.72 | 97.54 | 93.01 |
| MP4 | 15910943243 | 168623 | 50.94 | 96.95 | 91.47 |
| MP5 | 13158349550 | 145409 | 52.17 | 97.28 | 92.09 |

**Table S3** Water quality of full-scale CWs

| Sample site | NH_4_^+^- mg/L | NO_3_^-^-mg/L | TN mg/L | COD mg/L | TP mg/L | pH | ORP | DO | T |
| --- | --- | --- | --- | --- | --- | --- | --- | --- | --- |
| M1 | 0.50 | 7.73 | 9.13 | 20.8 | 0.3 | 7.54 | 176 | 9.32 | 18.3 |
| M2 | 0.22 | 1.52 | 3.42 | 15.81 | 0.36 | 7.54 | 172 | 11.97 | 18.9 |
| M3 | 0.15 | 6.24 | 8.27 | 11.65 | 0.35 | 7.38 | 180 | 6.4 | 17.5 |
| M4 | 0.21 | 1.04 | 1.05 | 10.82 | 0.31 | 7.44 | 167 | 6.61 | 15.8 |
| M5 | 0.15 | 1.47 | 1.76 | 5.87 | 0.34 | 7.21 | 166 | 4.19 | 16 |
| M6 | 0.32 | 2.14 | 2.65 | 3.83 | 0.36 | 7.64 | 154 | 8.49 | 15.3 |

**Table S4** Physicochemical properties of sediment

| Sample site | NH_4_^+^- mg/kg | NO_3_^-^-mg/kg | TN mg/kg | TOC mg/kg | pH | ORP | EC |
| --- | --- | --- | --- | --- | --- | --- | --- |
| M1 | 54.78 | 243 | 485 | 3.5 | 8.294 | -349 | 0.468 |
| M2 | 634 | 718.5 | 6870 | 53.8 | 7.838 | -305 | 0.738 |
| M3 | 167 | 206.65 | 2250 | 22.6 | 7.713 | -252 | 0.867 |
| M4 | 122.8 | 275 | 1480 | 16.4 | 8.065 | -293 | 0.701 |
| M5 | 96.55 | 563 | 3125 | 27.9 | 7.909 | -238 | 1.005 |
| M6 | 150.65 | 174.25 | 1915 | 8.4 | 8.19 | -276 | 0.326 |

| Topological parameters | MetaG-Plant | MetaG-Sediment | MetaG-Water | MetaT-Plant | MetaT-Sediment | MetaT-Water |
| --- | --- | --- | --- | --- | --- | --- |
| Edge | 194 | 172 | 211 | 150 | 81 | 77 |
| Node | 34 | 31 | 37 | 33 | 28 | 29 |
| Average degree | 11.412 | 11.097 | 11.405 | 9.091 | 5.786 | 5.31 |
| Graph density | 0.346 | 0.370 | 0.317 | 0.284 | 0.214 | 0.19 |
| Average clustering coefficient | 0.836 | 0.838 | 0.790 | 0.830 | 0.720 | 0.861 |

**Table S5** Topological properties of co-occurrence networks constructed from MetaG and MetaT datasets in different sample types.

**Table S6** Antibiotic concentration of full-scale CWs

| Antibiotic | Water ng/L | | | | | | | | | | | | Sediment ng/kg | | | | | | | | | | |
| --- | --- | --- | --- | --- | --- | --- | --- | --- | --- | --- | --- | --- | --- | --- | --- | --- | --- | --- | --- | --- | --- | --- | --- |
|  | MW1 | | MW2 | | MW3 | | MW4 | | MW5 | | MW6 | | MS1 | | MS2 | | MS3 | | MS4 | | MS5 | | MS6 |
| Trimethoprim | 5.28 | 1.32 | | 4.98 | | - | | - | | 1.24 | | - | | - | | - | | - | | - | | - | |
| Sulfamethoxazole | 1.16 | 1.66 | | 0.32 | | - | | - | | - | | - | | - | | - | | - | | - | | - | |
| Enrofloxacin | 3.06 | - | | 0.64 | | 1.00 | | 0.84 | | 4.74 | | 8757.89 | | 4989.71 | | 2825.66 | | 4803.79 | | 4214.86 | | 4434.50 | |
| Ciprofloxacin | 5.36 | 3.18 | | 4.66 | | 5.56 | | 1.90 | | 2.82 | | 17137.16 | | 6852.07 | | 14582.79 | | 8785.21 | | 25173.32 | | 7513.04 | |
| Ofloxacin | 14.10 | 6.76 | | 1.02 | | - | | - | | - | | 9295.92 | | 72230.34 | | 6224.36 | | 3379.70 | | 38779.70 | | 13820.77 | |
| Norfloxacin | 9.44 | 7.94 | | 10.52 | | 6.52 | | 4.60 | | 6.22 | | 5225.84 | | 5235.68 | | 5858.80 | | 5260.10 | | 3303.40 | | 8095.60 | |
| Lomefloxacin | 1.60 | 0.48 | | 0.08 | | - | | - | | 0.14 | | 3995.35 | | 3378.35 | | 2005.63 | | 2562.35 | | 1757.45 | | 4354.15 | |
| Oxytetracycline | 0.72 | 0.70 | | - | | - | | - | | 0.60 | | - | | - | | - | | - | | - | | - | |
| Tetracycline | 1.22 | 1.50 | | 1.00 | | 0.90 | | 0.94 | | 1.38 | | - | | - | | - | | - | | - | | - | |
| Anhydrochlortetracycline hydrochloride | 5.06 | 5.02 | | 3.30 | | 4.08 | | 3.38 | | 4.00 | | 682.50 | | 1149.54 | | 696.54 | | 722.07 | | 790.60 | | 592.61 | |
| Doxycycline | 1.84 | 4.44 | | 1.24 | | 0.84 | | 0.86 | | 1.30 | | 388.58 | | 486.92 | | 681.72 | | 295.85 | | 372.64 | | 356.57 | |
| Roxithromycin | 49.74 | 52.02 | | 49.46 | | 45.54 | | 45.52 | | 46.10 | | 1170.71 | | 5903.32 | | 2558.90 | | 1439.13 | | 3182.54 | | 2214.74 | |
| Lincomycin | 0.36 | 0.84 | | 0.22 | | 0.72 | | 0.96 | | 1.04 | | 518.10 | | 707.80 | | 908.95 | | 160.46 | | 377.68 | | 130.57 | |
| Azithromycin | 9.66 | 5.70 | | 12.62 | | 1.58 | | 2.88 | | 1.46 | | 1808.37 | | 2033.03 | | 770.64 | | 762.19 | | 1087.70 | | 969.26 | |
| Erythromycin | 27.26 | 0.00 | | 4.60 | | 152.52 | | 399.28 | | 165.60 | | 21934.57 | | 27026.77 | | 13071.16 | | 25894.31 | | 10423.84 | | 23312.51 | |
| Tylosin | 0.68 | 0.78 | | 0.62 | | 0.18 | | 0.14 | | 0.10 | | 488.21 | | 983.89 | | 360.62 | | 325.94 | | 282.00 | | 527.32 | |
| Fleroxacin | - | - | | - | | - | | - | | - | | 3527.07 | | 1997.89 | | 1037.39 | | 1158.32 | | 2135.12 | | 2747.08 | |
| Difloxacin | - | - | | - | | - | | - | | - | | 2575.56 | | 1611.37 | | 785.46 | | 616.77 | | 226.61 | | 572.52 | |
| Sulfacetamide | - | 2.58 | | 1.36 | | - | | - | | - | | 59.78 | | 2685.61 | | 4762.13 | | 1765.07 | | - | | - | |
| Thiamphenicol | 3.96 | 6.73 | | 4.46 | | 4.69 | | 9.16 | | 6.44 | | 680.01 | | 60.24 | | 1334.29 | | 35.10 | | 40.79 | | 655.38 | |
